# Similar Mechanisms Underlie the Detection of Horizontal and Vertical Disparity Corrugations

**DOI:** 10.1371/journal.pone.0084846

**Published:** 2014-01-03

**Authors:** Nirel Witz, Jiawei Zhou, Robert F. Hess

**Affiliations:** McGill Vision Research, Department of Ophthalmology, McGill University, Montreal, Quebec, Canada; CSIC-Univ Miguel Hernandez, Spain

## Abstract

Our aim was to compare sensitivity for horizontal and vertical disparity corrugations and to resolve whether these stimuli are processed by similar or radically different underlying mechanisms. We measure global disparity sensitivity as a function of carrier spatial frequency for equi-detectable carriers and found a similar optimal carrier relationship for vertical and horizontal stimuli. Sensitivity as a function of corrugation spatial frequency for stimuli of comparable spatial summation and composed of optimal, equi-detectable narrowband carriers did not significantly differ for vertical and horizontal stimuli. A small anisotropy was revealed when fixed, high contrast broadband carriers were used. In a separate discrimination-at-threshold experiment, multiple mechanisms of similar tuning were revealed to underlie the detection of both vertical and horizontal disparity corrugations. We conclude that the processing of the horizontal and vertical disparity corrugations occurs along similar lines.

## Introduction

Recently, we showed that there is a relationship between carrier spatial frequency and disparity corrugation spatial frequency [1]. Optimum disparity sensitivity occurs at low disparity corrugation spatial frequencies (<1 c/d) when the carrier spatial frequency is fixed at around 3 c/d. Above a disparity corrugation spatial frequency of 1 c/d, the optimum carrier spatial frequency is approximately 2.6x the corrugation spatial frequency. These results resolve what at first appeared to be a discrepancy between the results of a number of previous studies [2], [3], [4]. The consequence of this finding is that in order to validly compare how disparity sensitivity varies as a function of disparity corrugation, spatial frequency measurements should be done under conditions of comparable, and ideally optimal, conditions. The above carrier/modulator information is therefore important to ensure each disparity spatial frequency is measured under optimal conditions. Another requirement is that different disparity spatial frequencies to be compared should have a comparable number of cycles in width and height. When both of these requirements are met, the shape of the sensitivity function does not radically change, retaining its characteristic band-pass shape. [1].

It has been argued that the shape of the disparity sensitivity function is radically different in the low spatial frequency regions for vertical compared with horizontal disparity corrugations, the so-called stereoscopic anisotropy [5], [6], [7], [8]. Some have argued that the bandwidth of the disparity sensitivity function is reduced for vertical corrugations of disparity [7] others suggest a shift in the peak of the sensitivity function to lower disparity corrugation spatial frequencies for horizontal stimuli [5] and others have argued that the summation fields for vertical and horizontal corrugations are fundamentally different [9]. Indeed it has been suggested that the underlying mechanisms subserving the detection of horizontal and vertical disparity corrugations might be different; there being multiple channels for horizontal disparity corrugations but only a single channel for vertical corrugations [7]. Having previously established the optimal conditions for measuring sensitivity for vertical corrugations [1], we are in a good position to compare it to the sensitivity for detecting horizontal corrugations. We first set out to see if the same rules applied to the detection of horizontal corrugations as we had previously found for the detection of vertical corrugations; namely the carrier/modulator dependence.

## Materials and Methods

### 2.1. Apparatus

Psykinematix software v1.3.2 was used to generate and present all stimuli as well as record responses. A Macintosh computer running the Mac OS X version 10.6.8 ran the software while stimuli were presented on a 20-inch Dell Trinitron CRT monitor (40.5×30.5 cm). The display had a spatial resolution of 1024×768 pixels and the contrast resolution was 10.8 bits using the Psykinematix bit-stealing algorithm. The monitor was geometrically calibrated and gamma corrected using an Eye-One photometer (X-Rite i1 Display 2) using Psykinematix software v1.3.2. Disparity was generated by monocular displacements computed at sub-pixel resolution. Dichoptic presentation of the left and right eye images was achieved using CrystalEyes liquid crystal shutter glasses (RealD CrystalEyes 4). The monitor refresh rate was 120 Hz, so that each eye’s image was presented at 60 Hz.

### 2.2. Stimuli

For experiments measuring disparity thresholds in the fovea, a Gabor (modulator) disparity corrugation stimulus was used. The foveal stimulus consisted of circularly windowed, horizontal disparity corrugations of a band-pass luminance carrier. Peak luminance spatial frequencies of the carrier were from 0.5 to 10 c/d. The modulator disparity spatial frequencies tested were 0.25, 0.35, 0.5, 1, 2 and 4 c/d. However, cases where the carrier luminance spatial frequency was less than two times the modulator disparity spatial frequency were excluded because of sampling considerations.

**Figure 1:**
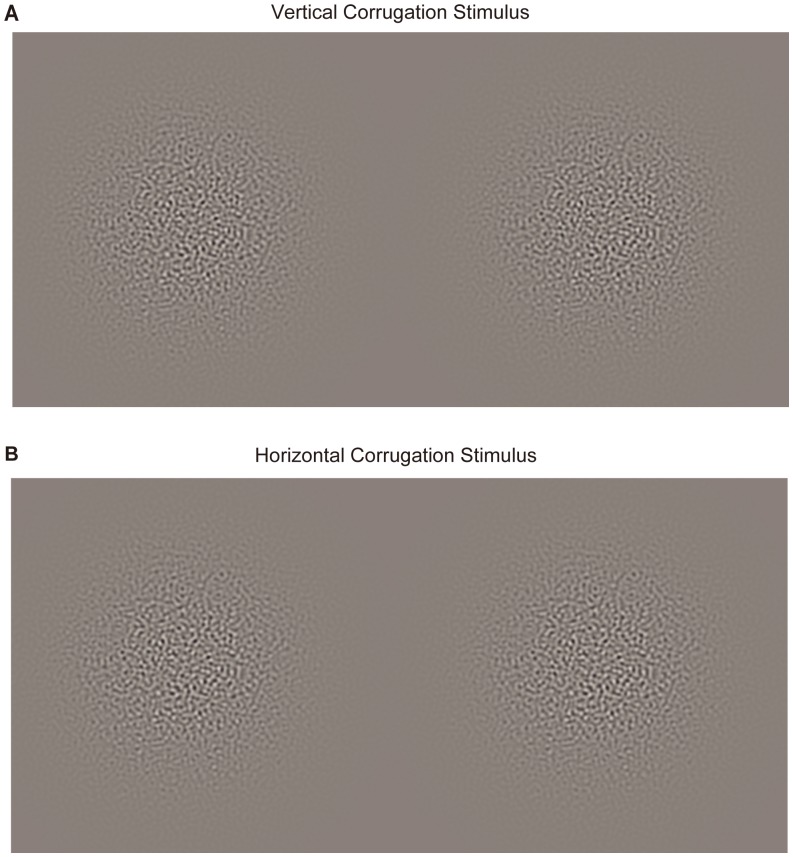
Stereo-pairs of the stimulus. Stimuli used for foveal global stereo sensitivity of vertical (A) and horizontal (B) corrugations. The foveal stimulus had a sigma of 9°. The luminance-defined carrier is band-pass noise whereas the stereo corrugation is a 1-D vertical (A) or horizontal(B) sinusoid for the foveal stimulus.

The foveal Gabor corrugation stimuli for the left and right eyes were generated by multiplying a luminance noise carrier by a 1-D vertical sinusoidal modulator. The carrier consisted of narrowband (1 octave, half amplitude, full bandwidth) filtered isotropic white noise set to 7 times its contrast detection threshold under all conditions. The global disparity sinusoidal corrugation was contained within a 2-D Gaussian spatial envelope (sigma was 2.2 cycles, see Figure 1) and presented abruptly in time (500 ms).

### 2.3. Observers

Two observers participated in all the foveal experiments of which one was naïve to the purpose of the experiments. Another naïve subject also participated in the test of the disparity sensitivity function for vertical corrugations using a carrier consisting of narrowband (1 octave) filtered isotropic noise set to 7 times its contrast detection threshold. All were experienced psychophysical observers and all had normal or corrected-to-normal visual acuity and stereo acuity. All studies were performed with the informed written consent of participants, were approved by the Research Ethics board of the Montreal Neurological Institute, and adhered to the tenets of the Declaration of Helsinki.

### 2.4. Viewing Conditions

The display was viewed at a distance of 45 cm, at which distance it subtended 47.5×36.5 degrees and a chin rest was used to locate the head.

### 2.5. 2-IFC Procedure

A staircase method was used to estimate the disparity threshold. The disparity amplitude was reduced after 2 consecutive correct responses and increased after 1 wrong response, corresponding to a criterion of 81.65% correct responses [10]. The initial disparity value (arc min) was randomly chosen (5±3 min arc). The reduction rate in disparity was 50% before the first reversal and 12.5% after the 1st reversal, while the increase rate was always 25%. Each session was terminated after 6 reversals and the threshold was computed from the mean of the last 5 reversals.

A two interval forced choice staircase technique was used to first measure binocular contrast thresholds (Michelson contrast) for broadband noise in which the detectability of the noise carrier was measured. The main experimental procedure was a temporal 2-IFC task where the subject had to indicate which temporal interval contains the target stimulus that was globally sinusoidally modulated in disparity. Subjects provided their responses using the keyboard arrows, left arrow for first interval and right arrow for second interval, during the post-stimulus interval. An auditory indicator was emitted at the beginning of each stimulus interval. The stimuli were presented abruptly for 500 ms. A fixation mark was present during the stimulus interval in the center of the display and subjects were asked to maintain their fixation during the whole presentation. Auditory feedback was given after each trial. The duration of the inter-stimulus interval was 500 ms. We ensured that thresholds were determined by disparity rather than any purely monocular displacement artifact by also measuring stimulus detectability without the stereo goggles under binocular viewing. These thresholds were always much higher than those obtained with the dichoptic presentation using the stereo goggles. The two thresholds were closest at 10 c/d, the highest carrier luminance spatial frequency used. Even under these conditions disparity provided the lower threshold.

### 2.6. 2×2 AFC Procedure

In order to assess whether thresholds were determined by a single or multiple underlying channels we use a procedure where discrimination of angular spatial frequency was measured at detection threshold. To measure discrimination at threshold, a 2×2 AFC paradigm was used in central vision in which one interval contained the disparity- modulated stimulus and the other just the noise carrier. Each presentation was 500 ms in duration. The subject had to answer two questions; firstly, which interval contained the stimulus (the detection response), and secondly, which of two different corrugation disparity spatial frequencies was presented (the discrimination response). Data were collected using a method of constant stimuli and the threshold was derived by fitting a Weibull function to the data. The statistical test for whether discrimination could be done at detection threshold (i.e., perfect discrimination) is discussed in Text S2.

Figure 1 shows examples of vertical (A) and horizontal (B) corrugation stimuli.

## Results

### 3.1. Foveal Measurements

**Figure 2:**
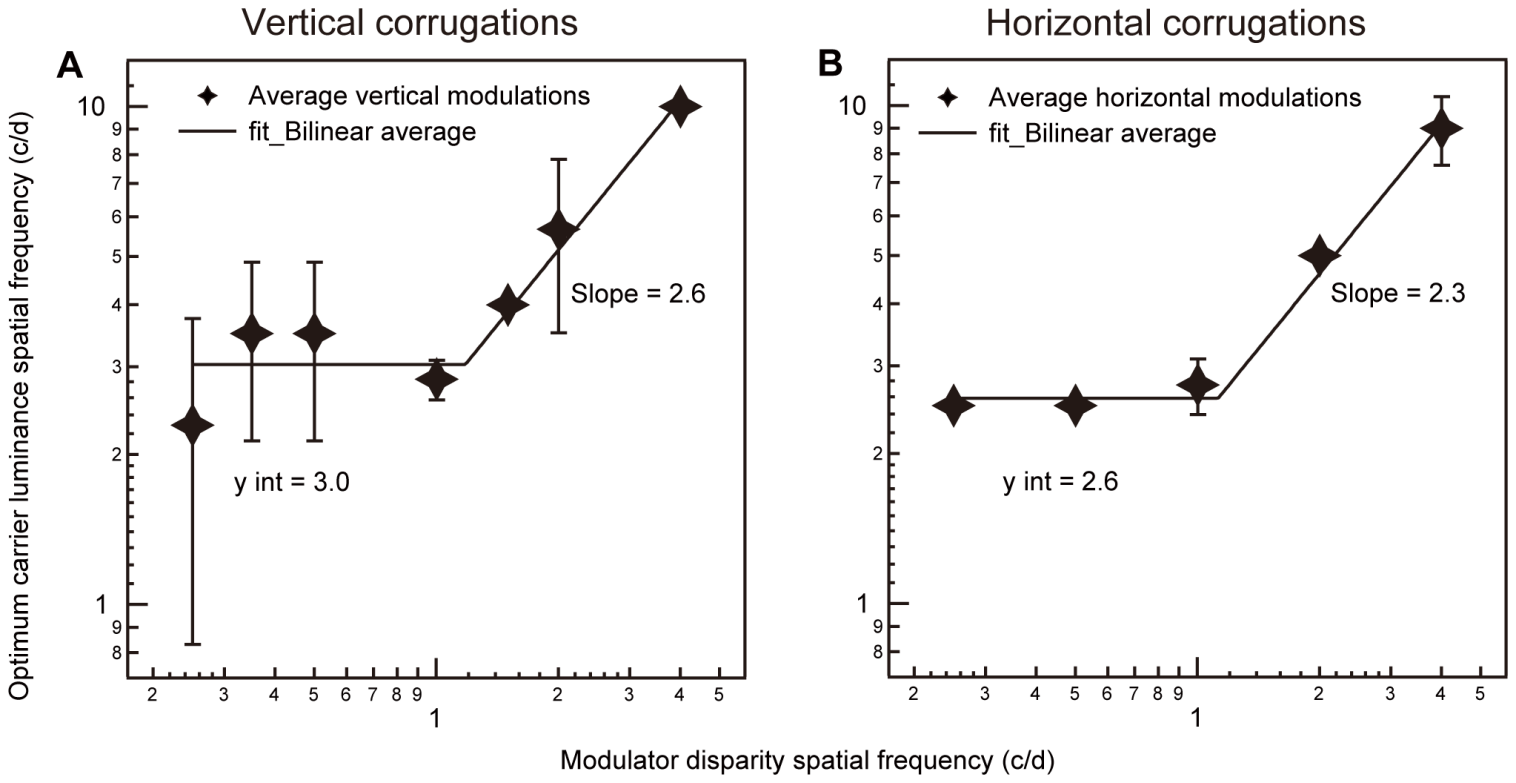
Optimum carriers for horizontal and vertical corrugations. Optimum carrier luminance spatial frequency (c/d) is plotted against corrugation disparity spatial frequency (c/d) as a function of the average across subjects including the standard deviation. Panel A represents optimum carriers across a range of vertically oriented sine-wave corrugations while panel B represents optimum carriers across a range of horizontally oriented sine-wave corrugations. The solid bilinear line in both panels indicates the line of best fit for the data.

Figure 2 shows averaged results for normal observers plotted in terms of the optimum carrier luminance spatial frequency as a function of the corrugation disparity spatial frequency. The averaged results for 6 normal observers tested from our previous work [1], with vertically oriented sine-wave corrugation stimuli are shown on the left (Fig. 2A) and the averaged results for 2 normal observers tested with horizontally oriented sine-wave corrugation stimuli are shown on the right (Fig. 2B). At the highest corrugation disparity spatial frequency (4 c/d), we could not make measurements for carrier luminance spatial frequencies above 10 c/d (maintaining our 7x contrast detection threshold criterion). Therefore the carrier luminance spatial frequency corresponding to the lowest disparity threshold (i.e., 10 c/d) was taken as the minimum. We found that a bilinear function provided a better fit (see Text S1 for the data compared with a linear function), even taking into account that the former has an extra parameter. The fits were done on linear/linear coordinates and are plotted here on log/log coordinates (slope of line has a zero intercept). For both vertically (Fig. 2A) and horizontally (Fig. 2B) oriented corrugations, the fitting (see Text S1) suggests that there is an optimal carrier for corrugation disparity spatial frequencies below 1 c/d and a different optimal carrier above 1 c/d. Below a corrugation disparity spatial frequency of 1 c/d, optimal disparity sensitivity is obtained for either a 3 c/d or 2.6 c/d carrier luminance spatial frequency in the case of vertical or horizontal corrugations, respectively. Whereas, for corrugation disparity spatial frequencies above 1 c/d, optimum disparity sensitivity is achieved for luminance carriers that are on average 2.6 times that of the disparity corrugation in the case of vertical corrugations (Fig. 2A) or are on average 2.3 times that of the disparity corrugation in the case of horizontal corrugations (Fig. 2B). The r-squares of the fitting in Fig. 2A and Fig. 2B are 0.94 and 0.99, respectively.

**Figure 3:**
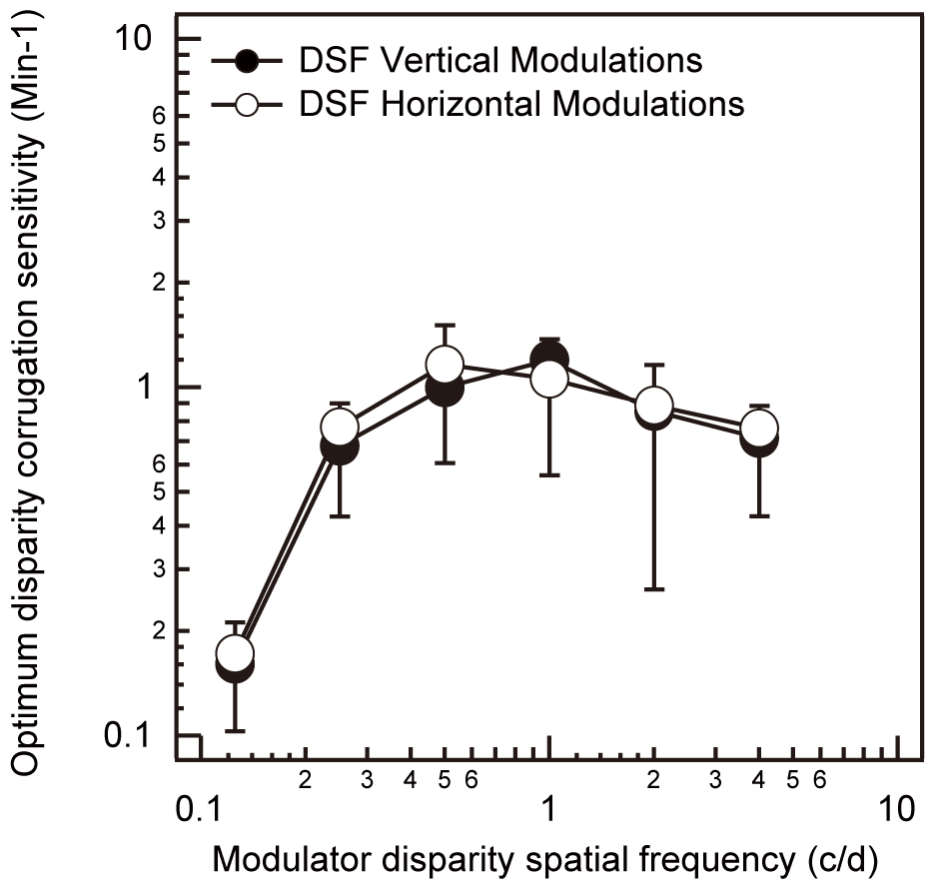
Optimized disparity corrugation sensitivity functions. Averaged optimum disparity corrugation sensitivity (min^−1^) is plotted against corrugation disparity spatial frequency (c/d) for a foveal stimulus whose number of spatial cycles did not vary with corrugation disparity spatial frequency. The black circles represent the disparity sensitivity function (DSF) for vertically oriented sine-wave corrugations with negative standard deviations. The white circles represent the DSF for horizontally oriented sine-wave corrugations with positive standard deviations.

Having derived the optimum carrier luminance spatial frequency for each corrugation disparity spatial frequency for both vertical and horizontal corrugations, we are in a position to measure the optimum relationship between disparity sensitivity and corrugation disparity spatial frequency for central vision (i.e., the foveal global disparity sensitivity function). To factor out any influence of spatial summation [9], [11], [12], [13] on the form of the foveal global disparity sensitivity function, we measured the relationship between both vertical and horizontal disparity sensitivity and corrugation disparity spatial frequency for a stimulus that was fixed in size in screen units but whose spatial frequency was changed by varying viewing distances. This ensured that all corrugation frequencies had the same number of spatial cycles in width and height. This result is shown in Figure 3 with sensitivity to vertical corrugations represented by black circles with negative standard deviations and sensitivity to horizontal corrugations represented by white circles with positive standard deviations. The contrast of all carriers was set to 7x their detection threshold. The carrier luminance spatial frequencies were set based on the bilinear fitted results in Figure 2, to make sure they are included in the 95% confidence bounds for both the vertical and horizontal conditions. In particular, the carrier luminance spatial frequency was set to 3 c/d for corrugation spatial frequencies in the range 0.125–1 c/d for both vertical and horizontal corrugation stimuli. At corrugation disparity spatial frequencies of 2 c/d and 4 c/d, the carrier luminance spatial frequency was set to 2.5x the corrugation disparity spatial frequency for both vertical and horizontal corrugation stimuli. These results show that for both vertical and horizontal corrugations the peak is broad (0.5–2 c/d) but there is still a clear low spatial frequency fall-off in sensitivity. We found no evidence of a stereoscopic anisotropy. (F(1,3) = 0.012, *p* = 0.92).

**Figure 4:**
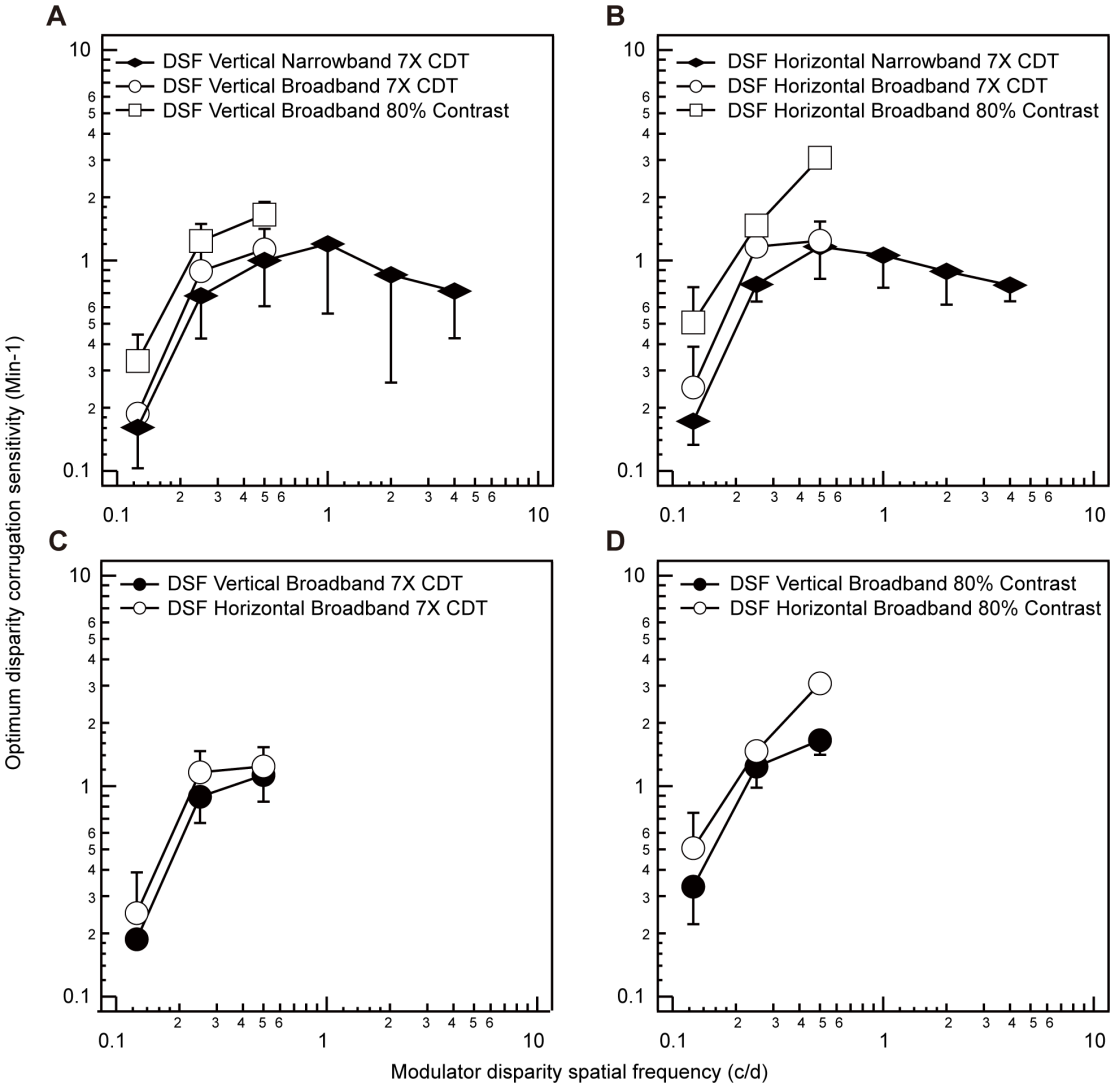
Disparity corrugation sensitivity for different carriers. Averaged optimum disparity corrugation sensitivity (min^−1^) is plotted against corrugation disparity spatial frequency (c/d) for a foveal stimulus whose number of spatial cycles did not vary with corrugation disparity spatial frequency. Disparity sensitivity functions (DSFs) are measured with the carrier consisting of either narrowband (1 octave) filtered noise set to 7 times its contrast detection threshold (7X CDT), broadband (6 octaves) filtered noise set to 7X CDT, or broadband (6 octaves) filtered noise set to 80% contrast for vertically oriented sine wave corrugations in panel A and horizontally oriented sine wave corrugations in panel B. In panels A and B, sensitivity to the narrowband noise carrier (1 octave) is represented by black diamonds with negative standard deviations and sensitivity to the broadband noise carrier (6 octaves) is represented by white circles (7X CDT) and white squares (80% contrast) with positive standard deviations. Panel C plots the disparity sensitivity function (DSF) for both vertical and horizontal corrugations whose carrier consisted of broadband (6 octaves) filtered noise set to 7X CDT. Panel D plots the DSF for both vertical and horizontal corrugations whose carrier consisted of broadband (6 octaves) filtered noise set to 80% contrast. For both panels C and D, the black circles represent sensitivity for vertically oriented sine-wave corrugations with negative standard deviations. The white circles represent sensitivity for horizontally oriented sine-wave corrugations with positive standard deviations.

While Figure 3 displays the disparity sensitivity functions for both vertical and horizontal corrugations using a carrier consisting of narrowband (1 octave) filtered isotropic noise set to 7 times its contrast detection threshold, we wanted to examine whether the shape of these functions would be affected by varying the bandwidth and contrast of the noise carrier, as all previous studies used broadband carriers set to a fixed high contrast [5], [6], [7], [8]. These results are shown in Figure 4 where sensitivity is compared as a function of both vertically oriented (Fig. 4A) and horizontally oriented sine wave corrugations (Fig. 4B) for a carrier consisting of either narrowband (1 octave) filtered noise set to 7 times its contrast detection threshold (7X CDT) as in Figure 3, a carrier consisting of broadband (6 octaves) filtered noise set to 7X CDT, or a carrier consisting of broadband (6 octaves) filtered noise set to 80% contrast. For both Figure 4 A and B, sensitivity to the narrowband noise carrier (1 octave) is represented by black diamonds and sensitivity to the broadband noise carrier (6 octaves) is represented by white circles (7X CDT) and white squares (80% contrast). The peak carrier luminance spatial frequency was set to 3 c/d for corrugation spatial frequencies in the range 0.125- 0.5 c/d for both broadband vertical and horizontal corrugation stimuli. The results in Figure 4A and B show that while carriers of higher contrast and broader bandwidths increase sensitivity at low to medium corrugation frequencies (Narrowband 7X CDT vs. Broadband 80% contrast: F(1,5) = 33.382, *p* = 0.002), the effects are similar for vertical (Figure 4A) and horizontal (Figure 4B) corrugations (the interaction of carrier and corrugation: F(1,5) = 4.347, *p* = 0.091). Figure 4 C and D show a comparison of sensitivity to vertical and horizontal corrugations under the three carrier conditions described above. Figure 4C plots disparity sensitivity functions (DSFs) for both vertical and horizontal corrugations for carriers consisting of broadband (6 octaves) filtered noise set to 7X threshold. Figure 4D plots the DSF for both vertical and horizontal corrugations for carrier consisting of broadband (6 octaves) filtered noise set to 80% contrast. For both Figure 4 C and D, sensitivity to vertical corrugations is represented by black circles and sensitivity to horizontal corrugations, by white circles. Carriers of higher contrast and broader bandwidth, as have been used in previous studies [5], [6], [7], [8] do result in superior sensitivity (horizontal vs. vertical in Figure 4D: F(1,2) = 17.705, *p* = 0.052) for horizontal corrugations (average across the above studies = ×5) but the magnitude of the anisotropy is not as large (present study = ×1.5) as previously reported.

## Discussion

In this study we show that sensitivity to horizontal and vertical disparity corrugations has a similar dependence on carrier spatial frequency. This leads to a re-examination of the relationship between both vertical and horizontal disparity sensitivity and corrugation disparity spatial frequency in the fovea using optimal carriers. Disparity sensitivity functions for stimuli equated for spatial summation [9], [11], [12], [13] and composed of optimum equi-detectable, narrowband carriers appear to be quite similar for vertical and horizontal corrugations. In other words, under these conditions we find little evidence for anisotropy. An anisotropy was evident for stimuli composed of broadband, fixed high contrast carriers although it was much less than previous reports suggested [5], [6], [7], [8]. These results find support in the previous results of Tyler and Kontsevich [9]who also showed there was no anisotropy for horizontal and vertical Gabor corrugations of 1 cycle in width and height. However, this does not imply that the underlying summation areas for corrugations of different orientation are identical because they show that the optimal summation area is more horizontally elongated for horizontal corrugations. Thus, even if the number of cycles are fixed across corrugation frequency, the absolute number may be crucial because Tyler and Kontsevich [9] showed that there was no anisotrophy when the number of cycles was fixed to 1 but there was an anisotropy when they were fixed to 4. The previous studies [5], [7], [8] that have reported an anisotropy for disparity corrugations have varied the number of cycles in the range 1–5.

Whatever the reason for the anisotropy in sensitivity for corrugations of the type used here what is clear is that the carrier/envelope spatial frequency ratio that results in optimal sensitivity is the same for horizontal and vertical disparity corrugations. This reflects the linkage between low-level local disparity detectors and higher-level global detectors responsible for the processing of these stimuli. For both vertical and horizontal disparity corrugations there is the same optimum carrier relationship, suggesting a comparable underlying detecting mechanism. The small difference in sensitivity that occurs (the anisotropy) for high contrast broadband carriers could suggest a subtle difference in the tuning and contrast response of the local disparity input.

Having shown that the shape and sensitivity of the foveal global disparity sensitivity function for vertically and horizontally oriented corrugations are similar, a remaining question is whether the processing of horizontal and vertical disparity corrugations is subserved by similar underlying mechanisms, more narrowly tuned for disparity. There is a previous suggestion that vertical corrugations are detected by a unitary mechanism [7] whereas the processing of horizontal corrugations is subserved by multiple mechanisms [7].

To resolve the issue of whether there is more than one channel subserving global disparity detection in the fovea for vertical corrugations, we had previously undertaken a 2×2 AFC detection/discrimination paradigm [1]. Subjects had to determine which of two presentations contained the disparity corrugations and then which of two corrugation disparity spatial frequencies it was. If stimulus disparity corrugation spatial frequency can be perfectly discriminated at its detection threshold, more than one unitary global disparity spatial frequency tuned mechanism must be involved [14], [15], [16] (see Text S2). We found evidence for more than one underlying mechanism for detecting vertical disparity corrugations; we now ask the same question, using an identical approach for horizontal disparity corrugations.

**Figure 5:**
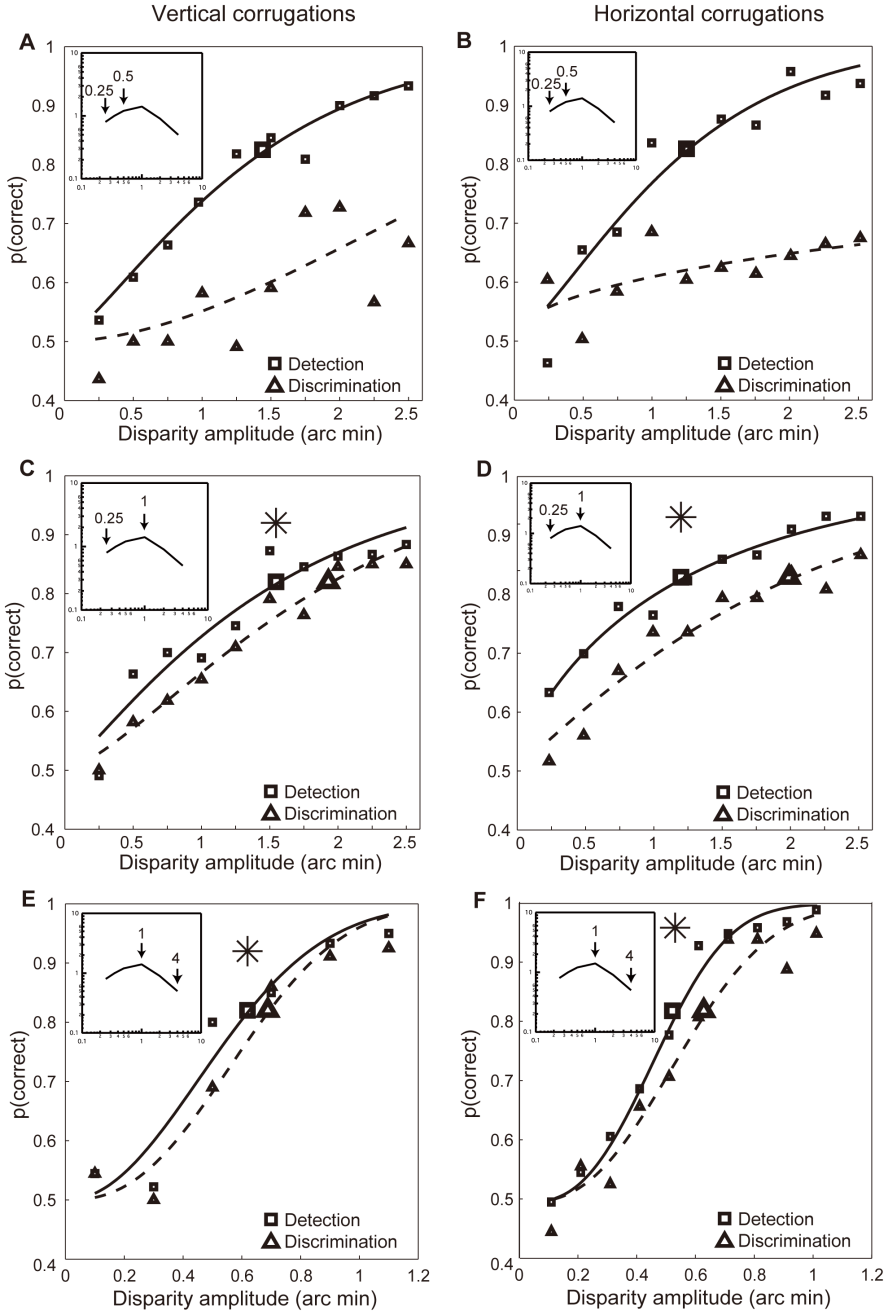
Discrimination of disparity corrugation spatial frequency at detection threshold. Foveal results from discrimination at detection threshold for 3 different pairs of horizontally oriented corrugation disparity spatial frequencies: 0.25 vs. 0.5 c/d (A), 0.25 vs. 1 c/d (B), and 1 vs. 4 c/d (C). The detection results from the 2×2 AFC paradigm are shown by small filled square stimuli and the discrimination results by small filled triangle symbols. The estimated threshold is shown by larger unfilled symbols. The ‘*’ indicates that perfect discrimination is possible at detection threshold as the thresholds for detection and discrimination are statistically indistinguishable (see Text S2).

Figure 5 shows the foveal discrimination results for different pairs of corrugation disparity spatial frequencies, each at their respective detection thresholds. For both vertical and horizontal corrugation stimuli, the results are similar. A corrugation disparity spatial frequency of 0.25 c/d cannot be discriminated from one of 0.5 c/d at threshold (Fig. 5 A and B), however a 0.25 c/d can be perfectly discriminated from one of 1 c/d (Fig. 5 C and D). Furthermore, a 1 c/d disparity corrugation can be perfectly discriminated from one of 4 c/d (Fig. 5 E and F). This suggests that there are multiple (a minimum of 3) channels underlying the overall foveal disparity sensitivity function for horizontal corrugations, in line with some previous reports [17], [18]. A similar result for vertical corrugations has also been shown [1].

The picture that emerges is that the mechanisms underlying the processing of horizontal and vertical disparity corrugations are quite similar. The overall sensitivity function (DSF), at least for equi-detectable, optimal, narrowband carriers is comparable. There appears to be multiple, more narrowly tuned mechanisms of comparable bandwidth underlying the DSF for both horizontal and vertical disparity corrugations.

## Conclusion

Optimal sensitivity for disparity corrugations depends on the carrier spatial frequency. To compare sensitivity across a range of disparity corrugation spatial frequencies it is important to do so for optimal, equi-detectable carriers ensuring also that corrugation spatial frequencies have comparable spatial summation. Under these conditions, sensitivity for horizontal and vertical corrugations is similar and the processing is subserved in both cases by multiple, more narrowly tuned disparity mechanisms.

## Supporting Information

Text S1 **Curve fitting for** **Figure 2** **.**
(DOCX)

Text S2 **The statistical test for discrimination-at-threshold.**
(DOCX)
