# Supplementary material for: Similar Mechanisms Underlie the Detection of Horizontal and Vertical Disparity Corrugations
Source: PLoS One. 2014 Jan 3;9(1):e84846. doi: 10.1371/journal.pone.0084846 (PMC3880320; doi:10.1371/journal.pone.0084846)
Supplement: Text S1 — Curve fitting for Figure 2 . (DOCX) [file pone.0084846.s001.docx]

**Text S1. Curve fitting for Figure 2.**

All the model-fitting procedures were implemented in Matlab using a non-linear least-square method and weighted by the standard error of each datum. The goodness-of-fit was evaluated by the *r^2^* statistic:

(A1)

An *F*-test for nested models (Hays, 1994) was used to statistically compare the models. For two nested models with *k_full_* and *k_reduced_* parameters, the *F* statistic is defined as:

$F(df_{1},df_{2})=\frac{(r_{full}^{2}-r_{reduced}^{2})/df_{1}}{(1-r_{full}^{2})/df_{2}}$,

(A2)

Where$df_{1}=k_{full}-k_{reduced}$, and$df_{2}=N-k_{full}-1$; *N* is the number of data points.

The average optimum carrier luminance spatial frequency versus modulator disparity spatial frequency curve (Figure 2) is fit by a bilinear function and statistically compared with a linear fit.

The bilinear fit assumes that the average optimum luminance spatial frequency (mean_OLSF_) remains consistent at low disparity spatial frequencies and linearly increases after one specific disparity spatial frequency (*D_sf_*). Thus, there are 2 free parameters, the intercept of the plateau part (i.e., ‘*b*’) and the slope of the linear part (i.e., ‘*a*’). The turning point is defined as the ratio of the intercept to the slope.

$$mean_{OLSF}=\frac{a\times Dsf+b}{2}+sign\left(x-\frac{b}{a}\right)\frac{a\times Dsf-b}{2}$$

(A3)

For the linear fitting procedure, there was only one free parameter, namely the slope ‘a’. The intercept was assumed to be 0.

$$mean_{OLSF}=a\times Dsf$$

(A4)

The results of statistical analysis are presented in Table A1, which indicate that the full model is significantly better (*P*=0.013) than the reduced model. Thus, we chose the bilinear fit.

Table S1. Statistical analysis for fittings of the average optimum carrier luminance spatial frequency versus modulator disparity spatial frequency curve.

| ***df_1_*** | ***df_2_*** | ***r^2^_full_*** | ***r^2^_reduced_*** | ***F*** | ***P*** |
| --- | --- | --- | --- | --- | --- |
| 1 | 4 | 0.936 | 0.646 | 18.006 | 0.013 |
