# Supplementary material for: Similar Mechanisms Underlie the Detection of Horizontal and Vertical Disparity Corrugations
Source: PLoS One. 2014 Jan 3;9(1):e84846. doi: 10.1371/journal.pone.0084846 (PMC3880320; doi:10.1371/journal.pone.0084846)
Supplement: Text S2 — The statistical test for discrimination-at-threshold. (DOCX) [file pone.0084846.s002.docx]

**Text S2. The statistical test for discrimination-at-threshold.**

The perfect discrimination hypothesis (i.e. that stimuli are discriminated as soon as they are detected) was more precisely tested using the probabilistic model of Watson and Robson (1981).

In this model, data were classified in four categories:

(1) *correct detection, correct discrimination*

(2) *correct detection, incorrect discrimination*

(3) *incorrect detection, correct discrimination*

(4) *incorrect detection, incorrect discrimination*

Following this model, which assumes that detection implies discrimination, we built a theoretical probability distribution for each level of modulation for each category:

*p(1) = 1 - p(2) - p(3) - p(4)*

*p(2) = g * (1 - b) * (1 - p_det_)*

*p(3) = g * b * (1 - p_det_)*

*p(4) = p(2)*

with *p_det_* the measured probability of detection of the stimulus and *g=0.5* the probability of guessing correct interval with no detection. *b* is the measured bias that each subject may choose one stimulus *vs*. the other in the case he was not able to discriminate it (for instance, in case one subject could not discriminate correctly, one could have a bias to more systematically choose the high frequency stimulus). b was typically between 0.4-0.6, see Watson and Robson, (1981) for more detail.

Then, instead of using the likelihood test, we tested the adequacy of our data compared to the theoretical distribution with a χ^2^-goodness-of-fit test (α=0.05). The null hypothesis is that the data fit the perfect discrimination model. Then the acceptance of the null hypothesis indicates that the conditions are “perfectly discriminated”. The acceptance of the null hypothesis is indicated by an asterisk in Figure 5.
